# The Failure of Blobology: fMRI Misinterpretation, Maleficience and Muddle

**DOI:** 10.3389/fnhum.2022.870091

**Published:** 2022-04-08

**Authors:** Stephen José Hanson

**Affiliations:** RUBIC (Rutgers University Brain Imaging Center), The State University of New Jersey, New Brunswick, NJ, United States

**Keywords:** fMRI, brain mapping, statistical approaches, networks, identifiability

Brain imaging has had enormous promise for many decades now. Magnetic resonance imaging was invented in the 1950s and rested on three Nobel Prizes and stunning engineering innovations. By the 1990s, SIEMENS, GE and a handful of other vendors were shipping scanners all over the world. Clinical applications provided immediate improvements in detection, surgical pre-screening and diagnostics, replacing behavioral tests, and risky pre surgical probes. MRI has been a boon to humanity. At the same time, basic research discoveries were not far behind, especially concerning dynamics of brain activity (BOLD-blood oxygen level dependency—effectively blood flow through the brain—which is a measure of neural transmission), but then it was clear that something else was missing: how do we make sense of what we are looking at? We are now all familiar with colorful brain activity in the New York Times and elsewhere, which often begs the question: what are these colorful blobs and what are their functions? Is there an obvious connection with the cognitive tasks typically used and brain blobs we record? Fundamentally, is there an epistemology of brain function that can be mapped from psychological tasks to anatomical areas? This question would dominate the field for the next 30 years.

There was an initial exuberance around brain imaging and many researchers felt they could finally start mapping brain function given the ease and access to human brain and behavior. Essentially, if you had access to a scanner, an IRB and willing volunteers, you were only limited by your imagination and stimulus construction (Posner and Raichle, 1994). Visual areas were initially mapped with basic psychophysical stimuli (checkerboard patterns, moving gratings...etc.). One unfortunate example of this initial over excitement was Wally Schneider a prominent Cognitive psychologist in the 1990s who was an early adopter of brain mapping and decided to bring visual cognitive psychology methods to MR, as reported in the *New York Times* in 1993:

*Dr. Walter Schneider, a psychologist who is using the technique*<*fMRI*> *to map human vision at the University of Pittsburgh, said, “We have, in a single afternoon, been able to replicate in humans what took 20 years to do in non-human primates.”*

I was one of the members of the advisory board for the McDonnell-Pew Foundation's Cognitive Neuroscience Program (which had invested millions of dollars into 10 Cognitive neuroscience centers throughout the world over a 10 year period—effectively creating the field of Cognitive Neuroscience), which ran from 1990 to 2000. In the early 1990s, Wally came by to give a talk and defend his claims while seeking funding. Between the various clashes going on during his talk between Wally and a conveniently Foundation-invited visual neurophysiologist (the Foundation enjoyed debate and liked to engineer it, if possible), I noticed he was using *t*-tests to test each voxel. And the *p* values were astronomically small (*p* < 10^−27^; e.g., Just et al., 1996). I asked “are you testing each voxel as a variable? If so, the false alarm rate must be enormous, or depending on the conditioning your miss rate must be enormous. Either case, what you are showing as a brain map can't be correct!” Wally responded, “Hmm. I see your point. Well, clearly we need some better statisticians!” Indeed, many folks thought better statisticians would be helpful and consequently this was the beginning of the rise of the University College London group (with K. J. Friston and R. S. Frackowiak) and *parametric brain mapping*. This was also the beginning of the normalization of brain mapping by an obscure Scottish Statistics Department and software (SPM due originally to K. J. Friston), which encapsulated the assumptions, the theory, and the models. Researchers now had tools, inferential statistics and software to analyze their data and to consequently create colorful blobs corresponding to statistical significance (red = 0.001, orange = 0.01, white = 0.05) a sunny side up egg with a red center! This was the rise of blobology.

## Blobology and the Search For Modularity in the Brain

Blobology naturally led to the question: what are the blobs? A natural answer is that they represented some type of localized function, some type of module, an encapsulated structure. Part of this question on locality, involves a statistical dimensionality problem (too many voxels). And the solution to this statistical problem was like most every other high dimensional data problem in astronomy, geology, or ocean mapping: just smooth, aggregate, and cluster, consequently reducing the effective number of variables and therefore degrees of freedom (there are other useful frameworks for controlling degrees of freedom as well, including false discovery rates, Euler fields, etc.,). This strategy could effectively shoehorn those *p* values to a Neyman–Pearson (often called *null hypothesis logic*) (Neyman and Pearson, 1933) standard range (0.001, 0.01, 0.05), with, of course, some loss of spatial resolution and with the increase of smoothed local topology, not necessarily apparent in the non-smoothed data. And for the next two decades, localization of function, no matter how illogical or absurd, became “brain mapping.” With a well-defined stimulus condition and an equally well thought through contrast, one could identify new functional brain areas every week^1^! This was dubbed “pure insertion.” It depended on strong assumptions, like additive independence of the brain function of the two conditions and therefore the lack of linear interaction (not to mention non-linear; Friston et al., 1996). But worse, it assumed the control condition, if simply “rest” was a tonic, homogeneous background. This turned out not to be true and in itself created a new field of studying “resting state networks”, (Biswal et al., 1995), in effect the background behavior of the brain. Although the absurdity of the discovery of a “face area” [an area of the fusiform gyrus that was somehow primarily responsible for face processing—fusiform face area (FFA) (Kanwisher et al., 1997)] wasn't sufficient reason to abandon the blobology program, subsequent years showed how bankrupt this phrenology (modularity) program had become. Because functional localization like the “face area” has no actual anatomical anchors in the brain, but was based on a procedure, using another set or all the existing face stimuli in the experiment to, unfortunately, create circular kind of evidence for where the face area was to be found in the fusiform gyrus (“localizer logic”, see Friston et al., 2010 who illustrates the dangers of localizers). Because the procedure could vary across labs, the brain coordinates of the FFA actually depended on the laboratory doing the studies (Berman et al., 2010; reanalysis of their data; LDA showed a significant “lab” predictor, this was also anecdotally confirmed)! There were NIH brain coordinates and MIT coordinates, typically differing in Z by 9–10 mm, and sometimes differing in the Y coordinate. The differences persisted from legacy lab to legacy lab with postdoc and graduate students trained in the FFA localization procedure from the lab in which they were trained. Other work using machine learning classifiers (support vector machines, neural networks, etc. Hanson et al., 2004; Hanson and Halchenko, 2008; Hanson and Schmidt, 2011) clearly showed that model-based conditional probability of brain tissue given the “face” stimulus, although might be found in fusiform gyrus, was actually more predictive in a number of other areas, including lingual gyrus, inferior frontal gyrus, inferior parietal lobule, etc. It was a cohort, a cluster, and, yes, most likely a “face network” as Haxby had predicted some time ago (Haxby et al., 2000). There is not sufficient space here to cover all the other FFA counter- evidence^2^. But suffice it to say, based on classifier evidence there is no “*... fusiform face area, a blueberry-sized region on the bottom surface of the posterior right hemisphere that responds significantly more strongly when people look at faces than when they look at any other stimulus class.”* (Kanwisher, 2006) Mathematically, this simply cannot be concluded from the GLM (unsupervised regression) or related tests.

Blobology also influenced the way networks were identified. In macaque, three areas of the brain (*f5* premotor, IPL, STS), representing pre-motor, attention, and joint perception of the same action, were dubbed the “mirror system.” Rizzolatti et al. (1996) called it a mirror system, because the same neurons responded when performing an action, like picking up a banana, as when viewing a video of another macaque picking up a banana. The neurons appeared tuned to perception-action whether the individual was performing it or watching it. As Rizzolatti and Craighero (2004) pointed out, using extremely clever experiments with dozens of control conditions:

“*In conclusion, the cortical mirror neuron circuit is formed by two main regions: the rostral part of the inferior parietal lobule and the ventral premotor cortex. STS is strictly related to it but, lacking motor properties, cannot be considered part of it. ”*

Now, this did not stop blobology from finding mirror areas by showing dancers to non-dancers and dancers to dancers and football players to non-football players... you get the idea. They (Iacoboni et al., 1999; Molnar-Szakacs et al., 2006) consequently found dozens more areas for the mirror system, because now they were easy to find—with careful thresholding. And often with the support of notable journals such as Science and Nature, the blobologists found pretty quickly that most of the brain was somehow involved in the mirror system. Rizzolatti, quite reasonably, argued that anatomical constraints must at least be the initial guidance on what the network might be, but this, like the face area, was going to end up being a network, though it has thus far only be dubbed a *system* (a network in waiting*)*, in contrast to a network, just a collection of areas. Nonetheless, as there were many valid ways to cook a chicken, there turns out to be many valid ways to find a network and some of them are in fact, functional (mathematical) not just structural (anatomical).

## The Network Muddle

Networks are formed by connections between regions of interest (ROIs). So the first problem was to find the right ROIs, and to find a set that did not seem arbitrary or contrived. Then time series could be measured over a so-called resting state, for example, when the subject is asked to do nothing but rest and relax. Bharat Biswal discovered resting states (Biswal, 2012) by a brilliantly simple experiment showing that seeding a resting state condition with one ROI time series of a previously executed motor task, would result in activity in all the other ROIs. In effect, the same motor network would pop out in the resting condition! It's as if the networks were latently present in the background, waiting to be summoned forth. Conferences, workshops, and journals became obsessed with resting state networks. Today the networks that arise are some of the most studied networks in brain science, and paradoxically with little agreement on what their function is at this point (but see Mastrovito, 2019)!

But how to measure the connectivity between the ROIs? The time series, once properly conditioned to remove drift, spikes, and irrelevant periodicity, could be correlated. But how much of the correlation is noise? Pearson correlation coefficient is subject to many kinds of maleficence, especially since we have no ground truth, not even simulation, that can guide us. Simulations in particular, due to over-simplying assumptions can be terribly misleading. For example, many assume white or gaussian noise as an additive source and are simply wrong, thus producing benchmarks that over-inflate goodness of fit and make differences small to statistically vanishing.

Worse was the unnecessary creative renaming and reinvention of known metrics, matrices, and methods. Perhaps the most egregious case was representational similarity analysis often termed RSA (Kriegeskorte et al., 2008). The idea of this “method” was to compute the “similarity” between brain ROIs and put them in a matrix to analyze their similarity structure. Sound familiar? In 1972, Shepard and Kruskal (1974) introduced novel theory and method to model rank-order data, in effect, inducing a distance space–interval/ratio data type of the similarity measures. This was multidimensional scaling which is used as input rank-order data (although it would work with any sort of distance or more generally association data from human similarity decision judgments. The theory of scaling was originally framed by Thurstone in the 1920s and was expanded and re-framed in the 1950s and 1960s. Suffice it to say that representational similarity analysis is a thinly veiled version of the original scaling framework and data theory (to be fair they do cite Shepard but then miss the point of finishing the analysis with either MDS, tree-fitting or clustering); Besides renaming matrices and then using multidimensional scaling, principal component analysis, independent component analysis, or the myriad of multivariate methods existent, this fad also spread to brain connectivity analysis. In this case, researchers use raw (or slightly conditioned) correlation matrices for analyzing brain connectivity. There are many problems with this strategy, not the least of which was arbitrary thresholding, inducing as many or as few clusters as one would like to find. Researchers began examining the correlation matrix directly, as if raw correlation matrix was itself a type of analysis rather than way-station to some valid testable inferential model! Although eliminating the thresholding issue, it turned the whole brain correlation connectivity exercise into a Rorschach test. Ok maybe I am too negative: What exactly is wrong with correlation or association or similarity?

1. *Correlations are associations not influences. consequently, they cannot orient edges*.
2. *Correlations fail to detect structure: most recent work in brain networks involving “functional connectivity,” often estimated using correlations of bold time series, have been shown to miss actual causal structure in known time series simulation tests* (Hanson and Glymour, 2010; Ramsey et al., 2010).
3. *Correlations are unstable. test-retest of resting state data in individuals reveals that correlation graph dissimilarity increases with sample size whereas bayes network estimated graph dissimilarity decreases with sample size as it should* (Hanson et al., 2018).
4. *Correlations across individual differences are statistically heterogeneous. resting state correlation matrices based on 235 rois over 70 subjects (pairwise) all tests fail a null hypothesis chi-square test, indicating that the correlations are rife with averaging artifacts* (Hanson et al., 2018).

It was simply astonishing that functional connectivity researchers using ROI correlations, did not also use latent analysis methods (principal component analysis or factor analysis or MDS) to model the correlation matrix, as most statisticians would have recommended. The patterns would have shown the same results and the model's assumptions would have been properly conditioned and rationally modeled even if there were millions of ROIs instead of a handful. Once again, It's like they read the first half of a narrative, fell asleep and missed the punchline (but see Reid et al., 2019).

## The Inference Muddle: Even Neyman and Pearson Would Have Been Confused

It became fashionable to attack brain imaging in the 21st century. We could start with the Dartmouth study that showed susceptibility artifacts in a dead fish, confusing the unwashed popular press to this day... but let's not. If you are confused about thermal noise, then you are probably living in it. And although there has always been a bit of self-doubt in neuroimaging folks, especially early on in panels with neuroscientists studying real neurons, the more serious inference troubles began when researchers began thinking independent samples meant something other than independent. Ironically, Nancy Kanwisher, the discoverer of the now dubious FFA, worked with a student, Ed Vul, who had noticed, with others, odd statistical anomalies (e.g., high correlations between brain activity and the walking speed with which subjects left the scanner room!) concerning brain imaging results, especially in social-neuroscience (Vul and Kanshisher, 2010). This problem is at least partly due to the weak correlation between theoretical constructs in much of social psychology and independent variables. Of course, having thousands or tens of thousands of independent variables (e.g. voxels) makes this problem more opportunistic. But, once again, this was nothing new. It was not unique to neuroimaging data and was well known in statistical theory, due to the work of statisticians like Efron and Tibsharani (1993) in regularization and machine learning (split half training/testing more specifically). In fact, once again this is not a serious flaw in brain imaging, but rather, and sadly, it was just unintentional maleficence. If the task is identification and categorization, you must keep test samples from being contaminated with training or model samples, and you can not use the training samples for testing! You must do N-fold cross validation. This is even more muddled once you realize all the fMRI time series are highly correlated, especially within an ROI (but that of course makes sense). Between ROIs the correlation is lower, but still driven by a large noise component in the BOLD signal, often used to define effective connectivity. Once again, like MVPA, which Haxby used to call “multi-VOXEL pattern analysis”, before he was reminded by his postdocs, that Multivariate pattern analysis (although even this phrase is redundant) was actually a thing. Kreigiskorte creatively introduced “double dipping” as a novel name for testing with the training set—i.e. *no cross-validation*. Better to use the original names with the original concepts—keeps things tidy.

## Replication Crisis? Or Individual Differences?

A well known kind of maleficence can occur due to individual differences in which averaging (smoothing) produces aggregates that misrepresent sub-group or individual differences. This problem is called Simpson's paradox. It is likely there is little homogeneity of activity throughout the brain, in that one group of subjects may have relatively sparse activity, and in another activity may be densely distributed. Worse, given the spatial distribution of activity in the brain, the topological properties may vary in terms of both the convexity and the amount of diffusion of activity (as in a Gaussian density or as an otherwise non-central set of spindles). In any case, individual differences in these patterns can lead to different “modes” which maximum likelihood averaging can result in non-representative group-wise clusters. Given different levels of smoothing at the hands of different research groups, very different densities of activity and locations can emerge. In some cases, there may be an easy way to mitigate this diversity: use better and more appropriate clustering methods—of which there are dozens of possibilities (Everitt, 1980 not just single linkage!). In other cases, the lack of a common topology of brain activity would make this challenging. In that case, individual differences are critical to understand—not just smooth into an aggregate brain pattern. The blobology maleficence was initiated on the assumption that inference in brain imaging should look like any other psychology task using 1-D measures like RT or accuracy. In that case, we would at least assume the BOLD distribution is substantially Gaussian. Unfortunately, it is not (Hanson and Bly, 2001; Wink and Roerdink, 2006).

## It's the Pipeline, Stupid! Bloblogy is Fine

Botvinik-Nezer et al. (2020) recently published a clever paper on something neuroimaging researchers already knew: Interpretations of fMRI results depend on decisions in the course of data workflow that can vary widely among researchers. In this study, using an identical data set (Botvinik-Nezer et al., 2019) and common preprocessing software, **70** independent groups of researchers produced surprisingly little agreement on **9** binary hypotheses about regions of the brain activated during a decision task. Although the number of active voxels varied from zero to tens of thousands over hypotheses, the overlap between group maps was very low. This primed the researchers' biases and top down visual clustering to identify map outcomes to be most consistent with the ROI hypothesis they may have favored. To be fair to the 70 groups, the measure of agreement=yes or no– is also a source of the divergence across the 9 hypotheses. If we assume, quite reasonably, that the assessment concerns the consistency of the whole hypothesis set, then the research groups had 2^9^, or 512, possible configurations to choose from. The probability of any given configuration by chance is (1/2)^9^ or 0.0002, which might also have fairly high variation in the distribution across all hypotheses—some well below and some above 1/2. Surprisingly, the actual hypothesis configuration they observed (0.371^*^0.224^*^0.229^*^0.329^*^0.843^*^0.329^*^0.057^*^0.057^*^0.057 = 0.0000003) is well below chance with only one case (#5,0.843) above chance. To be frank, this vector of outcomes is shockingly unlikely (700x below chance), and although apparently further supporting the conclusion of the authors, its anomalous nature urges one to scrutinize the entire exercise.

## So Some Good News!

But before we do that, it's worth clearing up at least one grievous misinterpretation due to this paper and others, that in some larger way, fMRI research is somehow fundamentally flawed or just so subjective as to be uninformative about brain structure and its mapping to cognitive and social function. *Of course this is not true*. Nor, in fact, do the authors imply that the lack of replication-based Neyman–Pearson (null hypothesis) testing across these 70 groups relates to the fMRI signal itself or to MR in general. Their clever demonstration highlights other problems with human brain mapping: in particular, I will argue, the presumed data structure, the lack of model-based analysis, and the focus on “real estate inference”. Neyman and Pearson would agree: part of the conclusions in this headline grabbing paper are merely based on conditioning from the specific hypotheses they gave the researchers (are there more than nine? Of course. Did they stack the deck? Maybe.). On the other hand, there is nothing wrong with what they did, since researchers make hypotheses all the time and expect activity or lack of activity in a specific ROI as a null hypothesis (although somebody must peek sometimes!). But if we simply look at the group agreement independent of the hypotheses—that is, we allow the research groups to effectively accept the null of each hypothesis or not—the agreement increases to 80%! The whole thing is a bit of a hat-trick. And in the larger context, this begs the question, again, why are we doing Neyman–Pearson hypothesis testing (null hypothesis logic) on 100s of thousands of voxels? This was the source of the astronomically low *p*-values we discussed in the beginning of this essay. What was the neuroscience, statistical theory, or logic that suggested this would be a productive thing to be doing? Botvinik-Nezer et al. (2020) focused in on this flaw, then prescribed better pipelines (fMRIPrep/BIDS?) and giving your data to a public repository to fix the problem. Nothing wrong with blobology, it's just the pipelines.

## How to Fix the Network Muddle?....Study Networks!

It is well known that the functional nature of an ROI depends on the cohort and context of other ROIs—in effect, a network (McIntosh, 2000). In fact, the problems revealed here are less likely or not likely at all to arise if the fMRI time series from ROIs (found by the general linear model or otherwise) are modeled as a network (although depending on the specific model, similar problems may arise). After all, the various hypotheses clearly involve multiple areas and, as any general linear model will reveal, are a larger set of cohort areas underlying various decision supporting functions (e.g., working memory, risk assessment, reward evaluation and so on; Poldrack et al., 2009). It is clear that the reliability of the fMRI result would increase if modeled as a network or a graph, not just because the data structure requires more weight of evidence, but because, with the correct type of graph search, individual differences can be more naturally accommodated.

Some 10 years ago, our fMRI collaboration group developed a graph (oriented edges) search method called (effective connectivity) **IMaGES** (Ramsey et al., 2010) for combining fMRI time series together across subjects while avoiding Simpson's paradox. This method takes as input the raw time series from all subjects/ROIs and finds the most likely graph (nodes and edges) that best represents all subjects as estimation is dynamically updated. Instead of averaging, it produces a graph that is a high probability compromise from constraint satisfaction over all sets of subject ROI time series, taking into account the heterogeneity of the individual differences in subjects and ROI time series. In the last decade network neuroscience has been on the ascendance, nonetheless the GLM and regularization for risk-minimization still forms the foundation of much of the research still being done.

Tangled up with this analysis muddle is this tenacious grip that specific identification of “real estate” has had this community from the beginning (Hanson and Bunzl, 2010). In effect, point estimation was considered to be the essential statistical problem and one that would eventually be informed by known ROIs that could be based on either theoretical or anatomical considerations, or both. In the 1990s, some 30 years ago, this seemed plausible. Now, given the advent of data science and the now conventional workflow involving full brain analysis and high-end computing, is a disastrous recipe for more malfiecance and muddle.

It has always been believed that transparency and wide public access to everyone's data sets would eventually clarify the functional and structural nature of the brain. Each research group could codify and share their pipelines, and all would in the end be valid and proper. Well, maybe. But, transparency and sharing is not going to necessarily increase reliability or validity of the result, since the individual differences are left as a random variable in the workflow and the underlying model is unspecified. This kind of muddle is an invitation to further confounding good science with bad science, good methods with bad methods. I propose, instead, it's time to move on, abandon the obsession with point estimates and blobology more generally, and embrace the ubiquitous networks in almost every neuroimaging study which will organically increase not only the reliability of the results, but their validity.

## Author Contributions

The author confirms being the sole contributor of this work and has approved it for publication.

## Conflict of Interest

The author declares that the research was conducted in the absence of any commercial or financial relationships that could be construed as a potential conflict of interest.

## Publisher's Note

All claims expressed in this article are solely those of the authors and do not necessarily represent those of their affiliated organizations, or those of the publisher, the editors and the reviewers. Any product that may be evaluated in this article, or claim that may be made by its manufacturer, is not guaranteed or endorsed by the publisher.

## Footnotes

^1^One anecdotal example of this problem floating around in the 1990s, was the peanut-butter and jelly study. The contrast was between high resolution pictures of peanut butter sandwiches and peanut butter and jelly sandwiches, thus through pure insertion, discovering the “jelly area” of the brain, and yes it was found in the fusiform gyrus!

^2^One telling example, In 1998 Kanwisher had a postdoc who submitted a poster to the McDonnell-Pew Foundation Cognitive Neuroscience workshop with not only “FACE” areas, but “PASTA”, “CARS”, “NUTS” etc..about 9 or 10 new areas. Another postdoc in her lab eventually ran obvious visual controls (bowl of wires and a bowl of nuts in the sense of bolts.. etc..) and almost all newly found areas disappeared except for the FFA. This is a testimony to the high quality folks in the Kanwisher lab, but at the same time reveals a deep modularity bias.
